# Supplement use among women experiencing hair loss

**DOI:** 10.1016/j.ijwd.2020.01.002

**Published:** 2020-02-05

**Authors:** Laura J. Burns, Maryanne M. Senna

**Affiliations:** Department of Dermatology, Massachusetts General Hospital, Boston, MA, United States; Department of Dermatology, Massachusetts General Hospital, Boston, MA, United States; Harvard Medical School, Boston, MA, United States

**Keywords:** Alopecia, Female pattern hair loss, Supplements, Vitamins

Hair loss of the scalp is an understandably distressing dermatologic concern for many women. Hair is deeply rooted in society’s measure of femininity, beauty, youth, and status in many cultures. Many are willing to go to extreme lengths to regain their hair and their self-confidence. At our specialty alopecia clinic, women have reported using onion juice, egg masks, cayenne pepper, and even Monistat yeast infection suppositories as scalp treatments.

It is axiomatic that women would turn to the $30 billion dietary supplement industry to self-treat hair loss. Perhaps this is, in part, due to extensive marketing on blogs, magazines, and social media, with promises of stimulating new hair growth and preventing future hair loss. A simple Google search for “hair loss supplements” yields a whopping 132 million results.

A retrospective analysis of patients with hair loss at our academic alopecia clinic showed that 81% of patients were female and had rates of reported supplement use higher than the national average. This was most notable among younger people (age 18–39 years), with 63% using supplements compared with the U.S. average of 40% (Kantor et al., 2016). Patients taking a hair-specific supplement, such as one labeled “Hair/Skin/Nails” or biotin, also had a higher number of total supplements used (4.5 supplements) compared with the overall study population (2.5 supplements).

Although commonly considered harmless, supplement use is not without risk. The U.S. Food and Drug Administration is not authorized to review supplements for safety and efficacy, and there are reports of inadequate active ingredients, microbes, heavy metals, and toxins found during compliance monitoring by independent organizations. Additionally, biotin, a ubiquitous hair loss supplement, has been shown to interfere with common diagnostic immunoassays, including thyroid stimulating hormones and troponins (U.S. Food and Drug Administration, 2018). This is particularly concerning given the high prevalence of thyroid disease and atypical myocardial infarction presentations in women.

Perhaps out of desperation for improvement or hope for a “natural” solution, patients often assume these risks despite the lack of benefit demonstrated in most randomized clinical trials of vitamin and mineral supplements. Micronutrient supplementation is recommended only for high-risk groups, such as folic acid during pregnancy or vitamin D in infancy (Manson and Bassuk, 2018). Otherwise, evidence does not exist for supplementation use in healthy individuals, whether in an attempt to improve general health or for hair growth.

The cost of supplement use extends beyond potential health risks and into women’s wallets. A single hair-specific vitamin can cost $50 to $100 per month, with costs totaling well over $1000 per year if multiple supplements are used. Hair loss is often chronic and progressive, and the expense compounds with years of use.

Dermatologists routinely struggle with the lack of effective hair loss treatments for women, and options are especially limited for those who are pregnant or would like to become pregnant. Yet, the time-old adage “can’t hurt, might help” should be used with caution, given the lack of regulatory oversight and interference with diagnostic laboratory assays. Physicians should strive to educate patients and overcome the widely disseminated marketing messages. The additional effort will potentially save this vulnerable population unnecessary medical work-up, thousands of dollars, and years of false hope.

## Financial disclosures

None.

## Funding

None.

## Study approval

The author(s) confirm that any aspect of the work covered in this manuscript that has involved human patients has been conducted with the ethical approval of all relevant bodies.

## References

[b0005] Kantor E.D., Rehm C.D., Du M., White E., Giovannucci E.L. (2016). Trends in dietary supplement use among U.S. adults from 1999–2012. JAMA.

[b0010] Manson J.E., Bassuk S.S. (2018). Vitamin and mineral supplements: what clinicians need to know. JAMA.

[b0015] U.S. Food and Drug Administration. The FDA warns that biotin may interfere with lab tests: FDA safety communication [Internet]. 2018 [cited 2019 August 14]. Available from: http://www.fda.gov/medical-devices/safety-communications/fda-warns-biotin-may-interfere-lab-tests-fda-safety-communication.

